# Global impact of COVID-19 on non-communicable disease management: descriptive analysis of access to FRAX fracture risk online tool for prevention of osteoporotic fractures

**DOI:** 10.1007/s00198-020-05542-6

**Published:** 2020-10-14

**Authors:** E. V. McCloskey, N. C. Harvey, H. Johansson, M. Lorentzon, L. Vandenput, E. Liu, J. A. Kanis

**Affiliations:** 1 Department of Oncology and Metabolism, Academic Unit of Bone Metabolism, Metabolic Bone Centre, Northern General Hospital, Centre for Integrated Research in Musculoskeletal Ageing, Mellanby Centre for Bone Research, University of Sheffield, Sheffield, S5 7AU UK; 2 Centre for Metabolic Bone Diseases, University of Sheffield, Sheffield, UK; 3 MRC Lifecourse Epidemiology Unit, University of Southampton, Southampton, UK; 4 NIHR Southampton Biomedical Research Centre, University of Southampton and University Hospital Southampton NHS Foundation Trust, Southampton, UK; 5 Mary McKillop Institute for Health Research, Australian Catholic University, Melbourne, Australia; 6 Department of Internal Medicine and Clinical Nutrition, Institute of Medicine, Sahlgrenska Academy, University of Gothenburg, Gothenburg, Sweden; 7 Region Västra Götaland, Geriatric Medicine, Sahlgrenska University Hospital Mölndal, Gothenburg, Sweden

**Keywords:** COVID-19, Fracture, FRAX, Non-communicable diseases (NCD), Osteoporosis

## Abstract

**Summary:**

The COVID-19 pandemic, and its management, is markedly impacting the management of osteoporosis as judged by access to online FRAX fracture risk assessments. Globally, access was 58% lower in April than in February 2020. Strategies to improve osteoporosis care, with greater use of fracture risk assessments, offer a partial solution.

**Introduction:**

The COVID-19 pandemic is having a significant detrimental impact on the management of chronic diseases including osteoporosis. We have quantified the global impact by examining changes in the usage of online FRAX fracture risk assessments before and after the declaration of the pandemic (11 March 2020).

**Methods:**

The study comprised a retrospective analysis using GoogleAnalytics data on daily sessions on the FRAX® website (www.sheffield.ac.uk/FRAX) from November 2019 to April 2020 (main analysis period February–April 2020), and the geographical source of that activity.

**Results:**

Over February–April 2020, the FRAX website recorded 460,495 sessions from 184 countries, with 210,656 sessions in February alone. In March and April, the number of sessions fell by 23.1% and 58.3% respectively, a pattern not observed over the same period in 2019. There were smaller reductions in Asia than elsewhere, partly related to earlier and less-marked nadirs in some countries (China, Taiwan, Hong Kong, South Korea and Vietnam). In Europe, the majority of countries (24/31, 77.4%) reduced usage by at least 50% in April. Seven countries showed smaller reductions (range − 2.85 to − 44.1%) including Poland, Slovakia, Czech Republic, Germany, Norway, Sweden and Finland. There was no significant relationship between the reduction in FRAX usage and measures of disease burden such as COVID-attributed deaths per million of the population.

**Conclusion:**

This study documents a marked global impact of the COVID-19 pandemic on the management of osteoporosis as reflected by FRAX online fracture risk assessments. The analysis suggests that impact may relate to the societal and healthcare measures taken to ameliorate the pandemic.

**Electronic supplementary material:**

The online version of this article (10.1007/s00198-020-05542-6) contains supplementary material, which is available to authorized users.

## Introduction

At the end of December 2019, a cluster of cases of pneumonia were reported by Wuhan Municipal Health Commission in China (https://www.who.int/news-room/detail/27-04-2020-who-timeline%2D%2D-covid-19). The outbreak eventually gave rise to the COVID-19 pandemic caused by the severe acute respiratory syndrome coronavirus 2 (SARS-CoV-2). While some countries appeared to control and minimise virus transmission, in many others the number of cases and, sadly, the number of deaths threatened to overwhelm healthcare and social systems. The fear that the same might happen elsewhere led to political, social and healthcare decisions to try to mitigate the impact, efforts which were spurred on by the declaration of a pandemic by WHO on 11 March 2020. While acknowledged as appropriate and necessary, it was widely recognised that the focus on COVID-19 would almost certainly lead to negative effects on the management of other diseases, particularly chronic non-communicable diseases (NCD), which frequently provided the greatest burden on healthcare systems, particularly in developed countries [1]. Documentation of this impact at a global level is complex, given the differences in health care systems, technology, coding methods and processes for monitoring such diseases.

Fractures that arise from osteoporosis are a long-recognised major cause of death and morbidity [2–4]. An estimated 2·7 million hip fractures occurred in 2010 worldwide [5]. In the EU, in the same year, 3.5 million new fractures were estimated to have occurred, comprising approximately 620,000 hip fractures, 520,000 vertebral fractures, 560,000 forearm fractures and 1,800,000 other fragility fractures [6, 7]. The cost of osteoporosis, including pharmacological intervention in the EU, was estimated at €37 billion, with two-thirds derived from the treatment of incident fractures and only 5% representing the costs of pharmacological prevention. The total cost including values of quality adjusted life years (QALYs) lost was estimated at €98 billion, a figure that is expected to rise to €121 billion in 2025. In the late 1990s, the World Health Organization (WHO) drew attention to the need to formulate a global strategy for the prevention and control of NCDs, including osteoporosis [8]. The subsequent work of the then WHO Collaborating Centre for Metabolic Bone Disease at the University of Sheffield led to the development and launch in 2008 of a freely available, fracture risk assessment tool (FRAX®, www.shef.ac.uk/FRAX) [2]. The tool is used to generate 10-year probabilities of fracture at the hip or major skeletal sites (hip, spine, humerus and distal forearm) using easily captured clinical risk factors, with or without the addition of femoral neck bone mineral density (BMD). FRAX has the advantage of being a global health care tool and has been widely adopted within clinical guidelines [9]. The current tool provides risk calculators for 66 countries and territories comprising well over 80% of the world population and is available in 34 languages. A recent survey found that it had been accessed from at least 228 countries or dependencies worldwide, with the USA being the source of the highest number of users [10]. As a global tool, the FRAX website provides an excellent opportunity to explore the impact of the COVID-19 pandemic on one aspect of care related to a NCD with significant global impact, osteoporosis and its related fracture burden.

## Methods

For this retrospective, descriptive analysis, we assessed worldwide and country/territory specific usage of the FRAX website by examining the number of sessions of website activity, and the geographical source of that activity, using GoogleAnalytics (https://analytics.google.com/analytics/web/). Briefly, GoogleAnalytics determines the user’s location from their IP address and counts each visit as a session. Currently, a session is defined as a group of interactions with the website that any one user can make within a 30-min time frame. The session number underestimates fracture risk calculations as more than one calculation can occur within the session. Data at the country level are described as accurate worldwide; it is believed that access via mobile devices or VPN can lead to inaccuracies in tracking the source, but usually within the country of origin (e.g. assigned to the wrong city).

A preliminary analysis of global FRAX access showed that levels of usage were stable from November 2019 to February 2020, so that we have restricted the main analysis and presentation of usage data to the 3-month period, February to April 2020, given that the WHO declared COVID-19 a pandemic on 11 March 2020. In addition, we undertook a visual comparison of FRAX access and usage within the same period in 2019. Data were exported from Google Analytics in XLSX format for further analysis. The analysis focussed on the number of sessions accessed from each country, and within each region/continent, in the 3 months of interest. For comparison across regions, the average changes in sessions were weighted by the number of sessions recorded in February within each country of the particular region. Finally, the changes in FRAX usage were correlated with markers of COVID-19 burden within each country, using data from the Worldometer COVID-19 database (https://www.worldometers.info/coronavirus/#countries; accessed 9th May 2020); these comprised the number of cases per million of the population, the number of deaths per million and, finally, the number of COVID-tests per million.

## Results

### Global changes

**Fig. 1:**
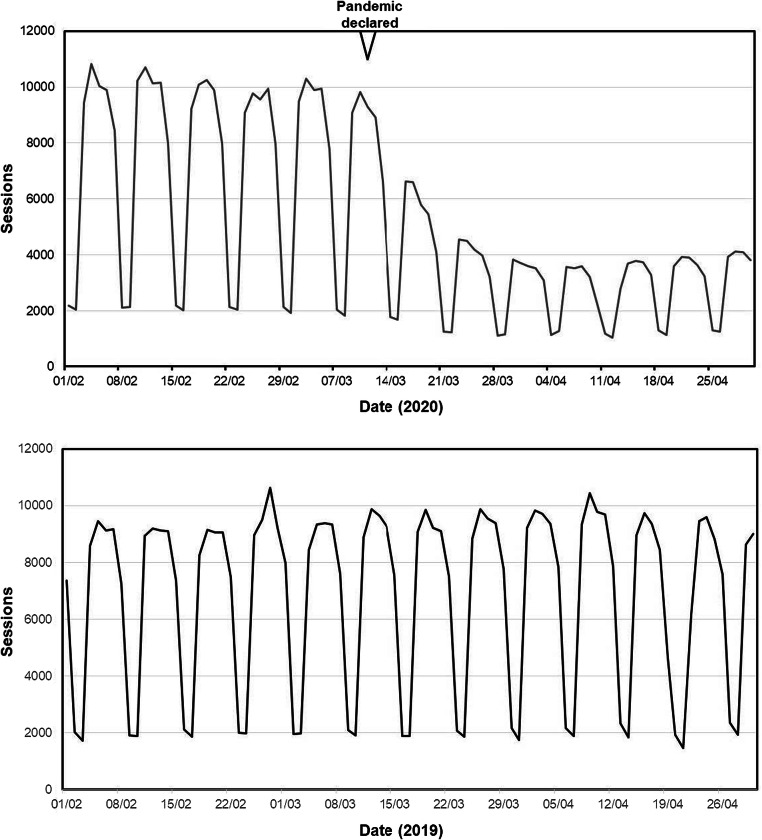
Daily number of sessions recorded on the FRAX online website during the months of February, March and April 2020 (top panel) and 2019 (bottom panel). The date of the declaration of the pandemic is also shown on the top panel. The cyclical pattern reflects increased usage on weekdays with lower usage at the weekends

The usages of the FRAX website for the periods of February–April 2019 and 2020 are shown in Fig. 1. The session rate remained constant throughout the observed period in 2019. During the 3-month period, February to April 2020 inclusive, the FRAX website recorded 460,495 sessions from 184 countries. The majority of these sessions (29.2%) arose from the USA. In February alone, an absolute number of 210,656 sessions were recorded, but this decreased to 161,986 in March and fell further in April to 87,853 sessions, representing decreases of 23.1% and 58.3% respectively from the number captured in February. As can be seen in Fig. 1 (top panel), the decrease in global usage started around the middle of March, consistent with the pandemic declaration, progressed during the second half of the month and remained low throughout the month of April.

Of the 184 countries, 139 had access or usage data for all 3 months. In order to make meaningful comparisons across countries, we confined further analysis to those countries with at least 100 sessions recorded on the FRAX website in February 2020. This cohort of 66 countries comprised 207,395 sessions in February, 159,466 sessions in March and 86,351 in April, representing 98.5%, 98.4% and 98.3% of the respective monthly totals. Results in terms of numbers of sessions and percentage changes from February through to April for all 66 individual countries are shown in Supplementary Table S1. Of these 66 countries, 15 were from Asia, 31 in Europe, 8 each in the Middle East/Africa (data only for South Africa) and Latin America and 2 each in North America and Oceania. The mean reductions in FRAX sessions in each region for March and April compared with February are shown in Table 1. A major difference was observed in the changes in FRAX usage with apparently much less marked reductions in Asia compared with Europe, Middle East/Africa, Latin America and North America. Oceania showed intermediate reductions.

**Table 1:** Percentage changes in online FRAX usage (sessions) for the 6 regions shown. The percentages were weighted by FRAX usage for each country within each region. The numbers in parentheses represent the range (minimum, maximum) of changes for countries within the region

		Percentage (%) decrease from February	
Region	Countries (*N*)	March	April
Asia	15	− 7.6 (− 38.3, + 23.4)	− 14.4 (− 78.9, + 232.8)
Europe	31	− 27.7 (− 74.5, + 17.4)	− 64.6 (− 96.5, − 2.85)
Latin America	8	− 16.7 (− 30.2, + 1.6)	− 61.4 (− 76.9, − 54.5)
Middle East/Africa	8	− 34.5 (− 44.9, − 11.6)	− 67.5 (− 84.3, − 26.0)
North America	2	− 22.0 (− 22.6, − 16.7)	− 59.0 (− 60.9, − 44.9)
Oceania	2	− 10.4 (− 13.1, − 9.0)	− 42.2 (− 64.5, − 31.4)

### Changes within regions

As expected, there was marked variability within each region (Table 1; Supplementary Table S1). For example, in Latin America, all 8 countries studied showed reductions greater than 50%, with the smallest reduction seen in Brazil (− 54.5%) and the greatest seen in Ecuador (− 76.9%). Within the Middle East/Africa region, only Qatar and Egypt showed reductions of less than 50%, with Egypt showing a reduction of only 26.0%.

**Fig. 2:**
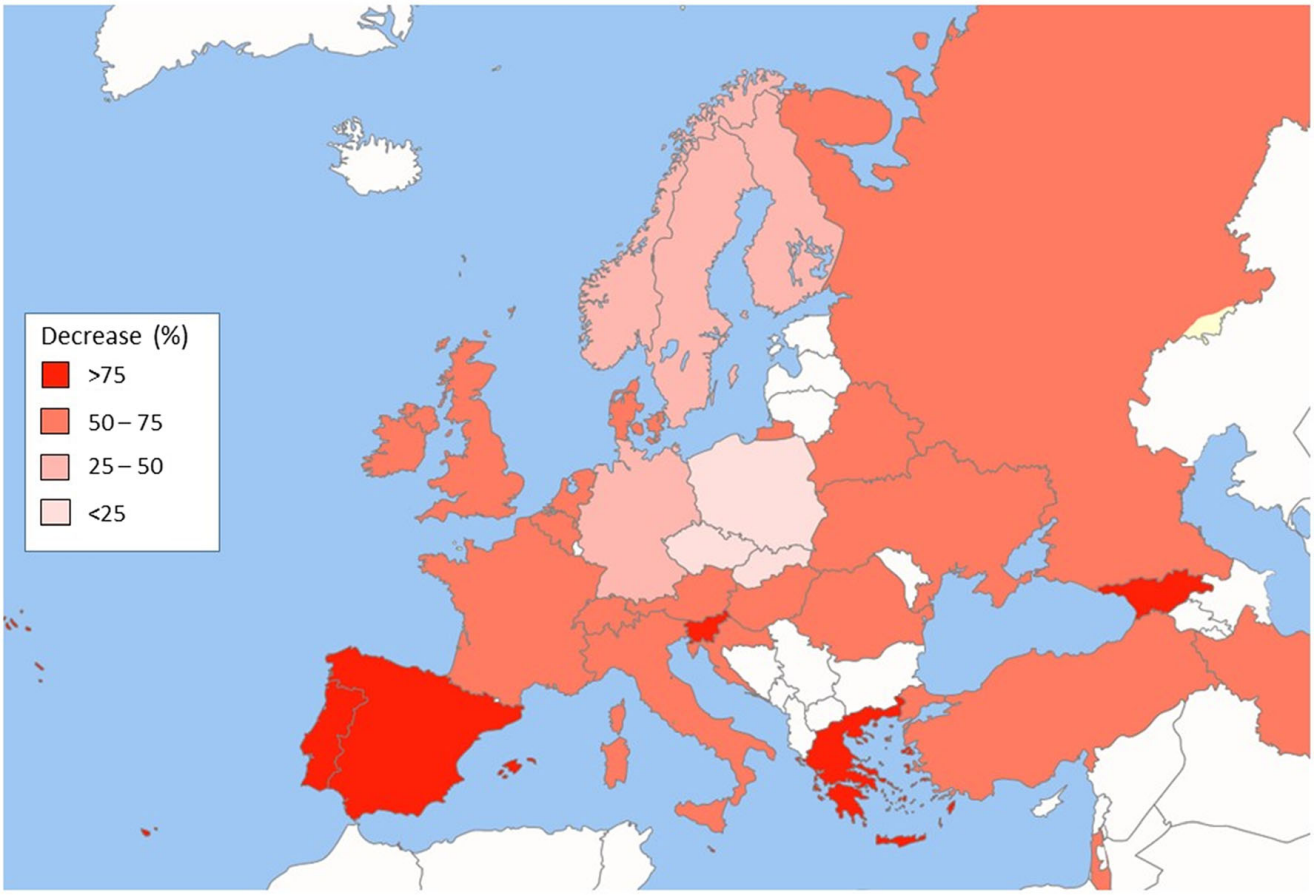
Map of Europe showing the reduction in FRAX usage between February and April 2020. Countries shown in white were not included in the analysis

In Europe, the majority of countries (24/31, 77.4%) showed reductions of at least 50% between February and April, with 6 of these (Spain, Portugal, Greece, Malta, Georgia and Slovenia) showing decreases of more than 75%. The 7 countries showing smaller reductions included Poland (− 2.85%), Slovakia (− 21.2%), the Czech Republic (− 22.6%), Germany (− 26.9%), Norway (− 31.7%), Sweden (− 41.4%) and Finland (− 44.1%) (Fig. 2).

In North America, proportional decreases in usage by April were greater for the USA than for Canada (− 60.9% and − 44.9% respectively). In Oceania, whilst the decreases in March were similar between New Zealand and Australia, the decrease observed between April and February was two-fold higher for New Zealand than for Australia (− 64.5% vs − 31.4% respectively).

**Fig. 3:**
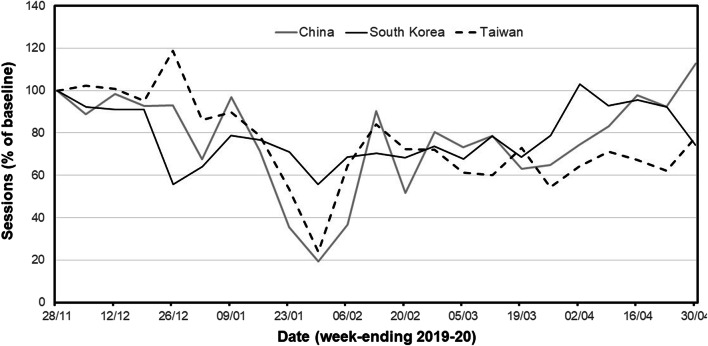
Weekly FRAX session numbers (expressed as a percentage of baseline values calculated from usage in November 2019) between December 2019 and April 2020 in the countries shown

The discordance in apparent changes was most marked in Asia (Supplementary Table S1). Here, only 4 countries (4/15, 26.7%) showed reductions of 50% or more in April compared with February; these comprised Thailand (− 52.1%), India (− 57.0%), the Philippines (− 78.9%) and Pakistan (− 63.6%). Intriguingly, 5 countries (33.3%) in the region showed an increase in FRAX sessions between February and April; Asia was the only region in which this occurred. The relevant countries/territories were Taiwan (+4.0%), Hong Kong (+23.5%), South Korea (+35.3%), China (+51.7%) and Vietnam (+232.8%). Subsequent analysis in these countries over a longer time frame, from the end of November 2019 until the end of April 2020, showed that the apparent improvement in FRAX usage had been preceded by earlier declines in FRAX session number (Fig. 3; Supplementary Figure S1). When expressed as sessions per month, the nadir for China and Taiwan occurred in January (− 39.9% and − 33.9% respectively, compared with November 2019), with nadirs for the other 3 countries occurring in February 2020 (− 56.4%, − 44.9% and − 32.6% for Vietnam, Hong Kong and South Korea respectively).

### Relationship of reduction in FRAX usage with markers of COVID-19 burden

There were very weak, non-statistically significant correlations between the impact of COVID-19 (cases, deaths and tests per million of the population) on FRAX usage, as judged by the reduction in usage between February and April. For the three measures, the correlation coefficients were 0.22 (*p* = 0.076), 0.21 (*p* = 0.09) and 0.20 (*p* = 0.112) respectively.

## Discussion

The direct impact of SARS-CoV-2 infection and the COVID-19 pandemic on morbidity, mortality and healthcare resource use across the globe has rightly been the focus of much research. NCDs have also featured prominently given the interaction between underlying health conditions and the likelihood of adverse outcomes during the infection [11–13]. Perhaps less frequently, but no less importantly, some have drawn attention to the detrimental impact that the focus on COVID-19 will have on the medium to long term management and outcomes for many common NCDs [14–17]. The present analysis is perhaps the first to quantify a very marked effect of the pandemic to reduce the assessment of fracture risk, a key component in the targeted initiation of preventative treatment to reduce the future burden of fractures. The reduction is substantial, averaging 58% but ranging up to 96%, and is truly global with two-thirds of the 66 countries/territories evaluated showing reductions by at least 50%.

The detrimental effect of the pandemic on osteoporosis and fracture risk is not only confined to a reduction in risk assessment [18]. Access to osteoporosis treatments that often require direct medical contact, such as intravenous or subcutaneous antiresorptives, are likely to be delayed or missed, raising the risk of further fracture particularly in the case of subcutaneous denosumab [19]. The opportunity provided by face-to-face contact via Fracture Liaison Services, at the time of or shortly after an incident fracture, or bone density services, to initiate treatment has more or less halted as secondary care has been diverted to COVID-19 care, or the risk of infection has been deemed too high. Furthermore, measures applied at city, regional and national levels around self-isolation and social distancing may well reduce exercise exposure and impair general health with a consequent increase in physical frailty, falls and related fractures.

The variation in reduction in FRAX usage is marked, and the reasons for this are likely to be complex. The most obvious observation is that the reduction in FRAX usage was much less marked in Asia than in other regions. The early and rapid response of China and its neighbouring countries/territories to the threat posed by SARS-CoV-2 has been much discussed. China itself commenced a mass quarantine of the city of Wuhan, the epicentre of the viral outbreak, on 23 January and the WHO declared the infection as a Public Health Emergency of International Concern (PHEIC) on 30 January. The close geographical proximity, lessons learned from the previous SARS and Middle East Respiratory Syndrome (MERS) pandemics in 2002 and 2015 respectively, and subsequent infrastructure investments, meant that an early combination of identification (based on symptoms or antigen testing), containment and contact tracing appeared to limit the spread of the disease and its impact on general healthcare. It is notable that the use of FRAX within the immediate region decreased but recovered quickly, suggesting that the measures capped the infection rate early before it overwhelmed the healthcare systems. In other Asian countries, for example in the Philippines, like many countries in Europe and beyond where some of the impacts have been substantial, the onset of action was delayed and occurred around the time of, or within days of, the declaration of a pandemic by WHO on March 11th. Our analysis notes an association between the later onset of measures to contain the outbreak and an apparently longer duration of suppression of FRAX access; the reasons for this are again likely to be complex, need further exploration and are beyond the scope of this report. For example, within Europe, the decrease in FRAX access in Poland appears to have been short lived, despite adopting apparently similar measures to neighbouring countries; the cause of this is unclear. However, what is clear is that the pandemic is having a significant impact on the management of a chronic NCD such as osteoporosis.

Our study has a number of limitations and strengths. The metric captured by GoogleAnalytics is sessions rather than individual calculations and is an underestimate of the latter. Over the 1-month period of April, there were approximately 123,000 fewer FRAX sessions than might have been expected. Bearing in mind that the session number underestimates the actual number of FRAX calculations by about 30% [20], then somewhere in the region of 175,000 patients were likely excluded from fracture risk assessment. If the measures adopted to address COVID-19 continue to impede osteoporosis care, then over a 3-month period more than 0.5 million patients would be excluded from assessment and a substantial proportion of those from necessary treatment. This is also an underestimate as many of the FRAX assessments worldwide are conducted on bone densitometers rather than through the web, activity which has largely ceased in many countries. As recognised by many, by the time this pandemic is over, a major challenge will remain to address and cope with the consequences of a huge backlog of NCDs. A major strength is that the study utilises a single metric captured via a single portal, the online FRAX fracture risk assessment tool, to compare the changes across countries with time. This contrasts with cross-country comparisons of, for example, the number of COVID-19 cases as the latter depends on political and healthcare policy and resources, and the integrity of mechanisms to categorise and count. The latter may have impacted on our attempts to correlate changes in FRAX usage with measures of COVID-19 burden.

Having observed and quantified the impact of the pandemic on a marker of NCD management, a number of questions arise, the most obvious one being how do we address the increasing backlog of these “non-essential” diseases in the short and longer term? We cannot simply ignore the fact that 71% of global mortality in 2016 was due to NCDs [21]. Within the field of osteoporosis, one potential answer lies with the FRAX tool itself. Many studies have examined its clinical utility since its launch in 2008; two systematic reviews have demonstrated the significant predictive ability of FRAX for future fractures, especially of the hip [22, 23]. In general, the incorporation of a measurement of femoral neck BMD results in higher accuracy than without BMD, but the additional value of BMD in all is modest [24]. It should also be recognised that even if the performance of FRAX is enhanced by the use of BMD tests, FRAX without BMD has a predictive value for fractures that is comparable to the use of BMD alone [25]. Several analyses have shown that patients identified at high risk of fracture by FRAX are responsive to osteoporosis treatments, including the recent study of screening by FRAX in the UK [26–29]. The availability and access to densitometry in many countries is low and access to largely secondary care-based facilities are further compromised or inhibited completely during the current pandemic [17]. Catching up with the backlog would be much enhanced by the assessment of fracture risk by FRAX in the absence of BMD in many patients, with the use of limited BMD resources targeted to those lying at or near intervention thresholds [24, 30]. The final major advantage in the current setting is that the assessment with FRAX can be undertaken remotely via telemedicine; indeed, future approaches could include embedding the FRAX risk assessment within electronic primary care records.

In summary, this analysis quantifies the impact of the COVID-19 pandemic and different approaches to its management on fracture risk assessment by the online FRAX tool. In many countries, the impact is large and persisting and it is highly likely that similar patterns are seen across many NCDs. Though not studied directly here, preparedness is likely to be one of the major drivers limiting the impact. Many lessons need to be learned for future national and international events impacting on health care resources.

## Electronic supplementary material

ESM 1 (DOCX 105 kb)
