# Supplementary material for: Global impact of COVID-19 on non-communicable disease management: descriptive analysis of access to FRAX fracture risk online tool for prevention of osteoporotic fractures
Source: Osteoporos Int. 2020 Oct 14;32(1):39–46. doi: 10.1007/s00198-020-05542-6 (PMC7556595; doi:10.1007/s00198-020-05542-6)

**Supplementary Materials**

**Table S1.** Changes in FRAX session number (expressed as a percentage change from the number recorded in February 2020) within regions and countries. Within each region, countries are ordered upwards from the greatest reduction seen between April and February.

|  |  |  | **Percentage change from February** | |
| --- | --- | --- | --- | --- |
| **Region** | **Country** | **FRAX Sessions in February 2020** | **March (%)** | **April (%)** |
| **Asia** | Philippines | 835 | -38.32 | -78.92 |
|  | Pakistan | 198 | -14.65 | -63.64 |
|  | India | 1174 | -20.27 | -56.98 |
|  | Thailand | 1807 | -25.95 | -52.08 |
|  | Kazakhstan | 132 | -28.03 | -47.73 |
|  | Malaysia | 528 | -6.06 | -35.8 |
|  | Sri Lanka | 136 | -31.62 | -22.79 |
|  | Singapore | 1489 | -1.61 | -15.51 |
|  | Indonesia | 461 | 10.85 | -11.93 |
|  | Japan | 4128 | -8.41 | -9.81 |
|  | Taiwan | 2088 | -0.77 | 3.98 |
|  | Hong Kong | 378 | 12.17 | 23.54 |
|  | South Korea | 615 | 23.41 | 35.28 |
|  | China | 1029 | 15.26 | 51.7 |
|  | Vietnam | 116 | 2.59 | 232.76 |
| **Europe** | Slovenia | 7736 | -60.33 | -96.54 |
|  | Georgia | 150 | -48.67 | -82.67 |
|  | Malta | 585 | -40.68 | -79.83 |
|  | Greece | 3126 | -42.64 | -77.35 |
|  | Portugal | 2563 | -39.37 | -76.04 |
|  | Spain | 7013 | -38.54 | -75.82 |
|  | Hungary | 2267 | -40.05 | -72.61 |
|  | Belarus | 157 | -24.84 | -72.61 |
|  | Croatia | 247 | -8.91 | -72.47 |
|  | Austria | 1817 | -31.59 | -72.37 |
|  | Turkey | 1035 | -43.29 | -69.76 |
|  | Belgium | 2010 | -34.73 | -69.1 |
|  | France | 3915 | -34.74 | -67.28 |
|  | Ireland | 1227 | -35.37 | -67.16 |
|  | Italy | 2366 | 1.14 | -65.34 |
|  | Ukraine | 637 | -27.63 | -65.31 |
|  | United Kingdom | 34426 | -23.33 | -63.56 |
|  | Israel | 1192 | -25.76 | -60.49 |
|  | Iran | 652 | -74.54 | -60.28 |
|  | Netherlands | 880 | -17.27 | -59.2 |
|  | Denmark | 155 | 17.42 | -58.71 |
|  | Switzerland | 2722 | -24.61 | -58.52 |
|  | Romania | 1025 | -49.46 | -57.95 |
|  | Russia | 9334 | -8.17 | -57.66 |
|  | Finland | 1902 | -13.67 | -44.06 |
|  | Sweden | 5245 | -7.91 | -41.35 |
|  | Norway | 552 | -14.49 | -31.7 |
|  | Germany | 832 | -24.4 | -26.92 |
|  | Czechia | 212 | 4.25 | -22.64 |
|  | Slovakia | 203 | -37.44 | -21.18 |
|  | Poland | 1792 | -31.31 | -2.85 |
| **Latin America** | Ecuador | 507 | 1.58 | -76.92 |
|  | Costa Rica | 320 | -18.13 | -73.75 |
|  | Colombia | 3201 | -24.49 | -67.07 |
|  | Mexico | 4108 | -16.21 | -65.34 |
|  | Chile | 817 | -30.23 | -64.5 |
|  | Argentina | 1263 | -26.44 | -61.44 |
|  | Peru | 286 | -25.17 | -57.34 |
|  | Brazil | 6962 | -10.86 | -54.45 |
| **Middle East and Africa** | South Africa | 172 | -25.58 | -84.3 |
|  | Kuwait | 194 | -44.85 | -72.68 |
|  | Saudi Arabia | 908 | -39.98 | -71.04 |
|  | United Arab Emirates | 362 | -30.94 | -70.72 |
|  | Jordan | 138 | -11.59 | -68.84 |
|  | Lebanon | 643 | -35.93 | -64.23 |
|  | Qatar | 109 | -23.85 | -49.54 |
|  | Egypt | 104 | -27.88 | -25.96 |
| **North America** | United States | 62046 | -22.64 | -60.88 |
|  | Canada | 8163 | -16.73 | -44.9 |
| **Oceania** | New Zealand | 1301 | -13.14 | -64.49 |
|  | Australia | 2702 | -9.03 | -31.42 |

**Figure S1**. Weekly FRAX session numbers (expressed as a percentage of baseline values calculated from usage in November 2019) between December 2019 and April 2020 in the countries shown.


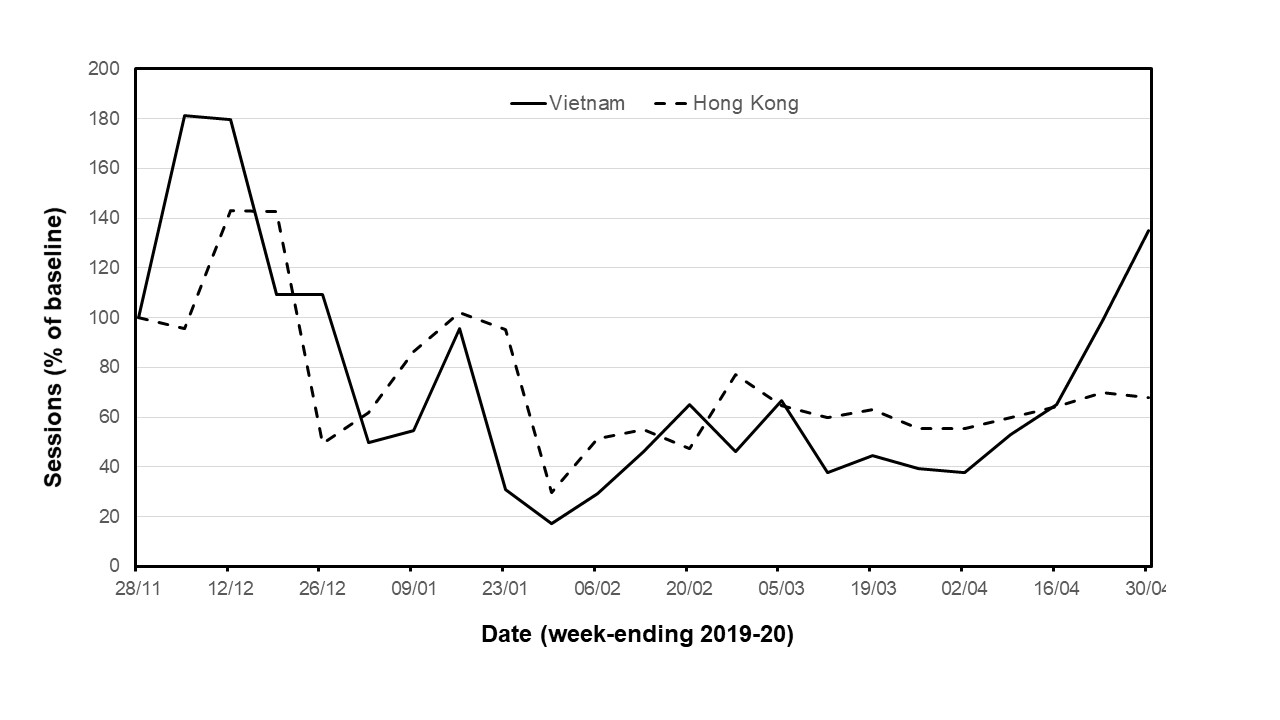

Supplement: Supplementary file 1 — (DOCX 105 kb) [file 198_2020_5542_MOESM1_ESM.docx]
